# High frequency oscillations in human memory and cognition: a neurophysiological substrate of engrams?

**DOI:** 10.1093/brain/awae159

**Published:** 2024-05-14

**Authors:** Michal T Kucewicz, Jan Cimbalnik, Jesus S Garcia-Salinas, Milan Brazdil, Gregory A Worrell

**Affiliations:** BioTechMed Center, Brain & Mind Electrophysiology laboratory, Department of Multimedia Systems, Faculty of Electronics, Telecommunications and Informatics, Gdansk University of Technology, Gdansk 80-233, Poland; Bioelectronics, Neurophysiology and Engineering Laboratory, Mayo Clinic, Departments of Neurology and Biomedical Engineering & Physiology, Mayo Clinic, Rochester, MN 55902, USA; BioTechMed Center, Brain & Mind Electrophysiology laboratory, Department of Multimedia Systems, Faculty of Electronics, Telecommunications and Informatics, Gdansk University of Technology, Gdansk 80-233, Poland; Department of Biomedical Engineering, St. Anne’s University Hospital in Brno & International Clinical Research Center, Brno 602 00, Czech Republic; Brno Epilepsy Center, 1th Department of Neurology, St. Anne's University Hospital and Medical Faculty of Masaryk University, member of the ERN-EpiCARE, Brno 602 00, Czech Republic; BioTechMed Center, Brain & Mind Electrophysiology laboratory, Department of Multimedia Systems, Faculty of Electronics, Telecommunications and Informatics, Gdansk University of Technology, Gdansk 80-233, Poland; BioTechMed Center, Brain & Mind Electrophysiology laboratory, Department of Multimedia Systems, Faculty of Electronics, Telecommunications and Informatics, Gdansk University of Technology, Gdansk 80-233, Poland; Brno Epilepsy Center, 1th Department of Neurology, St. Anne's University Hospital and Medical Faculty of Masaryk University, member of the ERN-EpiCARE, Brno 602 00, Czech Republic; Behavioural and Social Neuroscience Research Group, CEITEC—Central European Institute of Technology, Masaryk University, Brno 625 00, Czech Republic; BioTechMed Center, Brain & Mind Electrophysiology laboratory, Department of Multimedia Systems, Faculty of Electronics, Telecommunications and Informatics, Gdansk University of Technology, Gdansk 80-233, Poland; Bioelectronics, Neurophysiology and Engineering Laboratory, Mayo Clinic, Departments of Neurology and Biomedical Engineering & Physiology, Mayo Clinic, Rochester, MN 55902, USA

**Keywords:** network oscillations, intracranial EEG, local field potential, cognition, sharp-wave ripples, memory consolidation

## Abstract

Despite advances in understanding the cellular and molecular processes underlying memory and cognition, and recent successful modulation of cognitive performance in brain disorders, the neurophysiological mechanisms remain underexplored. High frequency oscillations beyond the classic electroencephalogram spectrum have emerged as a potential neural correlate of fundamental cognitive processes.

High frequency oscillations are detected in the human mesial temporal lobe and neocortical intracranial recordings spanning gamma/epsilon (60–150 Hz), ripple (80–250 Hz) and higher frequency ranges. Separate from other non-oscillatory activities, these brief electrophysiological oscillations of distinct duration, frequency and amplitude are thought to be generated by coordinated spiking of neuronal ensembles within volumes as small as a single cortical column. Although the exact origins, mechanisms and physiological roles in health and disease remain elusive, they have been associated with human memory consolidation and cognitive processing.

Recent studies suggest their involvement in encoding and recall of episodic memory with a possible role in the formation and reactivation of memory traces. High frequency oscillations are detected during encoding, throughout maintenance, and right before recall of remembered items, meeting a basic definition for an engram activity. The temporal coordination of high frequency oscillations reactivated across cortical and subcortical neural networks is ideally suited for integrating multimodal memory representations, which can be replayed and consolidated during states of wakefulness and sleep. High frequency oscillations have been shown to reflect coordinated bursts of neuronal assembly firing and offer a promising substrate for tracking and modulation of the hypothetical electrophysiological engram.

## Teaser

Kucewicz *et al.* review the role of high frequency gamma and ripple oscillations in human memory and cognition, and propose that these fast activity bursts track coordinated firing of neuronal assemblies across the brain during the formation and recall of memory traces.

## Bridging neural oscillations and neuronal spiking in memory and cognition

Intracranial electrophysiological recordings and stimulation have provided unprecedented access to study the neural activities that underlie the most complex and abstract functions of the human brain.^1-8^ Local field potential (LFP) recordings reflecting the activity of neural populations associated with memory and cognitive functions can be either oscillatory or non-oscillatory. The emergence of high-density macro-, meso- and micro-electrode arrays now enables recording the wide range of various LFP activities.^9-12^ Still, probing specific electrophysiological activity recorded at various scales to determine their origins remains a major challenge,^13-15^ as is mapping these to particular processes underlying our cognition.

**Figure 1:**
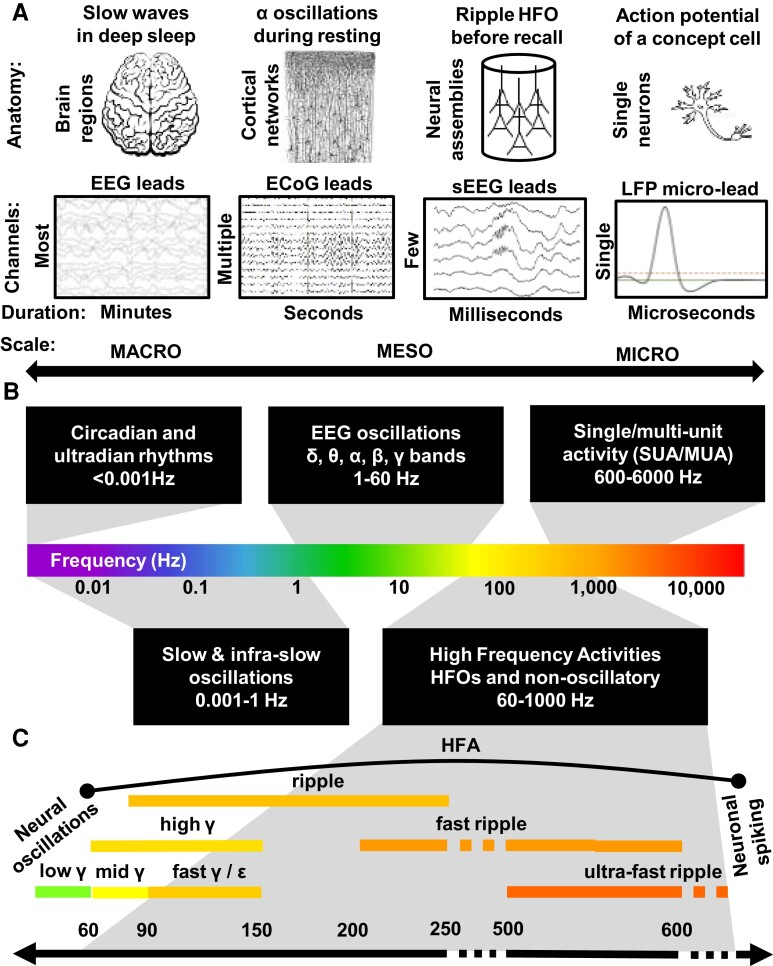
**High frequency local field potential activities bridge neural oscillations with neuronal spiking across the large scale of brain electrophysiology**. (**A**) Four examples of electrophysiological activities recorded across a range of anatomical scales show gradually increasing focality and spatiotemporal granularity of the source neural populations, assemblies and single cells. (**B**) The span of local field potential (LFP) activities extends from slow rhythms to fast oscillations and waveforms of individual action potentials. (**C**) High frequency activities comprise a variety of overlapping frequency subranges used to classify physiologically high frequency oscillations (HFOs) and other LFP activities. ECoG = electrocorticography.

At the high resolution end of the electrophysiological activity spectrum, neuronal action potential spiking, also known as the single unit activity, has been linked to mental representations of abstract concepts and proposed as a building block for our thinking and declarative memory.^8,16-18^ These single neuron spiking activities are confined to extracellular field potentials sampled on a micrometre scale from electrode contacts in the immediate vicinity of a spiking cell.^19^ On the other end of the spectrum (Fig. 1, top), there are LFP oscillations in the classic EEG spectrum generated by coordinated synaptic currents of large neural populations. The oscillations are traditionally classified into distinct frequency bands, commonly referred to as brain waves or rhythms, which are thought to be generated at different scales of neural organization. Low frequency oscillations of the delta or theta bands engage larger volumes and spatially extended neural networks, whereas higher frequency rhythms in the gamma bands are more local and confined to more specific neuronal ensembles.^20,21^ Between the two ends of this spectrum, linking the low ranges of the classic EEG bands (<60 Hz) and the high ranges of detecting the neuronal action potentials (>600 Hz), there is a wide frequency span of electrophysiological activities, including high frequency oscillations (HFOs) and other non-oscillatory sources of spectral power (Fig. 1, bottom). The HFOs may serve as a bridge to link the ‘building blocks’ of single neuron spiking with large-scale LFP activities reflected in neural network oscillations.^22^

In this review, we will first define the terms for various types of electrophysiological activities captured by extracellular LFP recordings and the vast frequency spectrum between EEG oscillations and neuronal spiking. Various terms that are used for similar LFP activities in overlapping frequency ranges have been a matter of recent controversy in dissociating sharp-wave ripples from HFOs or broadband power increases.^23,24^ These previous reviews were focused on the lower ranges of the high gamma and ripple frequency spectrum, treating HFOs in the ranges beyond 250 Hz as predominantly related to the pathophysiology of epilepsy. Here, we focus on the HFOs across the wide frequency range between the EEG oscillations and neuronal single unit activity. We will review the basic physiology of HFOs with reference to the other high frequency LFP activities with particular focus on human studies to support the proposed terms and definitions and to provide background for the neuropsychological theories of neuronal assemblies and engrams posed in the title question.

This will set the scene for discussing what is known about the roles of HFOs in memory and cognition. Several recent studies have reported HFOs in the ripple frequency and beyond to be associated with memory encoding, reactivation, recall and consolidation across hippocampal-cortical networks.^25-33^ We will conclude by discussing the title proposal that HFOs can provide electrophysiological substrates of engrams, reconciling the single neuron research of abstract concept representations, spatiotemporal dynamics of high frequency LFP activities across the brain and the most recent advancements in the engram research. Finally, we will conclude with a prospective use of HFOs to track large-scale dynamics across widespread networks of connected neural assemblies during memory and cognitive functions.

## Classification and definitions of electrophysiological activities in the high frequency spectrum

### HFOs are classified across overlapping frequency ranges based on physiological properties

Given the wide variety of LFP activities recorded in the high frequency range between the classic EEG bands on one end and the neuronal spiking on the other (Fig. 1), it has been a major challenge to determine distinct boundaries and names in this previously uncharted territory. Over three decades of animal and human studies, describing both physiological and pathological, oscillatory, non-oscillatory, induced/evoked and spontaneous phenomena, have produced nomenclature and definitions that are not fully consistent and standardized in one unified view. A consensus statement has been published recently by a representative group of researchers defining sharp-wave ripples in the context of other high frequency LFP activities in humans, non-human primates and rodents.^23^ The statement addresses the key problems in the field related to signal processing and exclusion of artefacts, methods for detection and analysis of LFP ripples, anatomical localization in the hippocampus and neocortex and relationship to other physiological and pathological discharges in the spectrum. Some of these problems were also discussed in a special volume of reviews dedicated to the HFOs.^24,34,35^ Both the recent statement and the previous reviews pertained mainly to the high gamma and ripple frequency ranges, aiming to separate them from other pathological discharges and non-physiological artefacts. Oscillations beyond these ranges were mostly treated as related to the pathophysiology of epilepsy.^36^

Figure 1 (bottom panel) summarizes the proposed classes of distinct high frequency activities, including both oscillatory and non-oscillatory sources of LFP spectral power. The lower end of the HFO spectrum is dominated by several classes of gamma and ripple frequency ranges, which are highly overlapping within a span extending from 60 to 150 Hz. On the higher end, the frequency boundaries for fast and ultra-fast ripples and their relationship with and influence from neuronal spiking activities^37,38^ have not been clearly defined.

There has been more in-depth research into the contribution of neuronal spiking to spectral LFP activities recorded in the gamma and ripple frequency ranges.^39,40^ The lower frequency ranges (>60–250 Hz) are more clearly charted and classified on the spectrum based on their physiological phase coupling with oscillations in the classic EEG bands like the theta rhythm.^41-43^ Hence, three frequency subbands were proposed within a wider gamma range: low (30–90 Hz) and fast/epsilon band (∼90–150 Hz), which are separate from the overlapping ripple range (∼140–220 Hz).^35^

Focusing on the oscillations, distinct classes have been separated based on the underlying neural mechanisms of their generation. One of the first such distinctions was made in rodent hippocampus between ripples occurring in the 140–200 Hz range, which were associated with sharp-wave bursts of neuronal spiking and relatively high amplitude of oscillations visible in the raw signal, compared to fast gamma/epsilon oscillations of lower frequencies (100–130 Hz), which were not associated with the sharp-wave bursts but shared common neuronal mechanisms with the ripples.^44^ A later study confirmed that these two classes of HFOs are quantitatively distinct but share similar neuronal networks and mechanisms.^45^ Both studies found that the ripples (140–220 Hz) and fast gamma/epsilon (90–140 Hz) oscillations had different anatomical localizations in the hippocampal subfields and the connected neocortical areas.

In general, there is ample evidence for different types or classes of HFOs based on their anatomical locations, neural substrates and mechanisms of generation.^35^ Separating these based on frequency boundaries into distinct gamma, ripple and higher frequency ranges appears challenging and cumbersome because the boundaries are highly overlapping (Fig. 1).

### Defining human HFO types in specific frequency ranges and anatomical localization

It would seem from these original studies in the rodent hippocampus and mesial temporal neocortex that ripples and gamma HFOs should be easily distinguishable based on either the frequency range (higher for ripples), mechanism of generation (e.g. presence of a LFP sharp-wave) or anatomical location (e.g. hippocampal subfields). In the human hippocampus, however, ripples with the greatest amplitude were detected in 80–140 Hz frequency range,^31,46-48^ which overlapped with the high gamma range (70–150 Hz). Sharp-wave bursts have not been commonly used for detection of ripples or separation from high gamma LFP activities in these original or subsequent studies since they were conducted in people with epilepsy, who have epileptiform sharp-waves (i.e. interictal epileptiform spikes) often accompanied by a HFO. Differentiating pathological epileptiform sharp-wave transients and physiological sharp-waves and the associated HFOs is a challenge.^25-28,31-33^ In these cognitive studies, therefore, the only criterion used to distinguish ripples from high gamma or epsilon oscillations is the anatomical localization in the hippocampus or, more recently, in its CA1 subfield. But since gamma and ripple HFOs share common cellular mechanisms,^44,45^ they are virtually impossible to separate without the main distinguishing sharp-wave feature.

Ripples are also recorded outside of the hippocampus proper or the connected mesial temporal lobe structures. One of the first reports of neocortical ripples in rats described them as ‘spike-and-wave discharges’ with oscillations in the fast ripple frequency range.^49^ Neocortical ripple oscillations were later found to be synchronized with the hippocampal ripples, especially during sleep following learning,^50^ congruent with their proposed roles in hippocampal-cortical transfer of information related to memory consolidation.^51-53^ Recent studies in humans report similar co-occurrence of hippocampal and cortical ripples during sleep and cognitive tasks.^26,29,30^

Furthermore, ripples are commonly recorded both in the epileptic and non-epileptic hippocampus and neocortex,^32,46,48,54-58^ and it remains a matter of controversy whether distinct classes of physiological and pathological HFOs can be separated by frequency, amplitude or any other characteristic. The same term ‘ripples’ and especially ‘fast-ripple’ has thus been used to describe a pathological class of events in the hippocampus and the neocortex without a clearly established relationship to the physiological hippocampal-cortical interactions.

Altogether, classifying various gamma, ripple and fast ripple HFO types based on anatomical location, states of sleep or wakefulness, physiological or pathological roles is an even more cumbersome task in human studies, where there is less mechanistic insight than in the animal models.

### Using frequency range instead of a definite class is a more replicable alternative

What we are left with is a general category of HFOs with specific labels used for approximate frequency ranges, as presented in Fig. 1. Naming frequency ranges with explicitly specified low- and high-end boundaries provides a more replicable and robust approach than attempting to identify a distinct class of events across animal and human studies. For instance, instead of labelling a given class of oscillations like ripples in humans based on the sharp-wave ripple complexes in rodents, one can objectively define the ripple frequency range that was used. For instance, stating that: ‘oscillations were detected in a ripple frequency range (80–150 Hz)’ would be a more objective and replicable alternative to ‘ripples were detected between the 80–150 Hz frequency range’. The former only claims that the detected events were actual oscillations as opposed to other non-oscillatory sources of spectral power in this frequency range but claims no particular class of LFP activity. The latter explicitly claims that the detected events were ripples, presumably corresponding to the sharp-wave ripples in rodents, as opposed to other high gamma/epsilon oscillations.

Definite statements about an HFO type like ripples should only be made if supported with enough evidence,^23,24,35^ for example, concurrent detection of sharp-wave transients and micro-electrode recordings of single unit spiking^25,34,59,60^ that would correspond to the patterns reported in rodent electrophysiology. For this reason, we suggest using the general term HFOs without making connotations to any specific class or frequency range of oscillations—pathological or physiological, unless explicitly stated. Since the gamma, ripple and fast ripple oscillations share common neuronal mechanisms of generation, it seems appropriate to refer to them with the general term HFOs in particular frequency ranges. Emerging computational tools^61,62^ and analysis methods^63^ for automated and objective classification of various HFO types offer a promising future direction for defining and differentiating distinct types of HFOs.

Having established the basic definitions, we will now turn to the neural correlates of HFOs and set the scene for addressing the title question about their role in human engram processes.

## Neuronal assembly origins and mechanisms of HFO generation

### HFOs are generated by coordinated spiking of local neuronal assemblies

Early studies of HFOs in epilepsy patients using macro- and micro-contact electrodes showed that oscillations recorded in the ripple and fast ripple frequency ranges are very local. Given that an individual ‘fast ripple’ HFO (250–600 Hz) can be detected on a single micro-wire but not on any of the neighbouring ones in the same bundle, it was estimated that these are generated within 1 mm^3^ of neural tissue.^47,64^ The origins of these fast ripple HFOs would thus be confined to a volume as small as a single cortical column, as in the case of other micro-scale electrophysiological discharges recorded both in people with epilepsy and in some cases patients with chronic pain and no history of epilepsy.^65^

**Figure 2:**
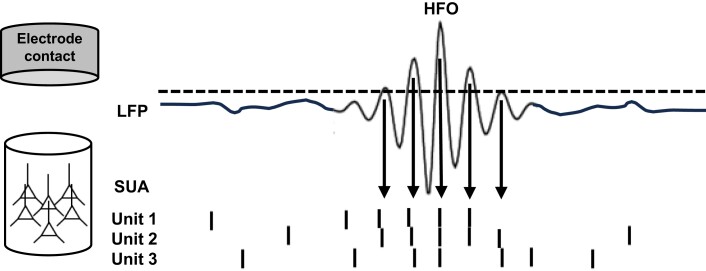
**Simplified model of generation and detection of high frequency oscillation bursts**. Individual bursts of oscillations in the local field potential (LFP) signal are recorded from electrode contacts that are proximal to the source generator of a neuronal assembly. The assembly is composed of a network of connected neurons, which in case of fast ripple high frequency oscillations (HFOs) can be organized within a volume and cytoarchitecture of a single cortical column. Individual assembly neurons fire action potentials—measured as single unit activity (SUA)—with more or less coordinated timing of their spiking. If the spiking is temporally aligned it drives deflections in the LFP signal, compared to the periods when it is not coordinated in time and the LFP signal is flat. Repeated discharge of this coordinated assembly firing results in a burst of an oscillation with deflections corresponding to cycles associated with the temporally aligned spiking (arrows). Frequency of the emergent oscillation depends on the interval period between the coordinated spiking. If a given unit fires a burst of action potentials at intervals as small as the refractory period limit (approximately 2.5 ms), the emergent oscillation can reach a peak frequency of 400 Hz. Notice that individual units in a given assembly can skip cycles of an oscillation because other units are firing at that time. Hence, the emergent frequency of an HFO can exceed the limit of maximum burst spiking frequency of any one unit, which explains the ‘in-phase’ and ‘out-of-phase’ mechanisms of HFO generation.^66,67^ The outcome of this coordinated SUA is detected in the LFP signal as an oscillation upon crossing of an arbitrary amplitude threshold of detection (dashed line), which is usually set above 3 standard deviations from the signal mean. Duration of a given burst can thus be determined between the detection thresholds of a given burst with at least four cycles, lasting approximately between 50 and 100 ms.^33^ Hence, these bursts can be treated as discrete binary events, much like the spiking activity,^22^ centred around the peak amplitude at a given frequency.

**Figure 3:**
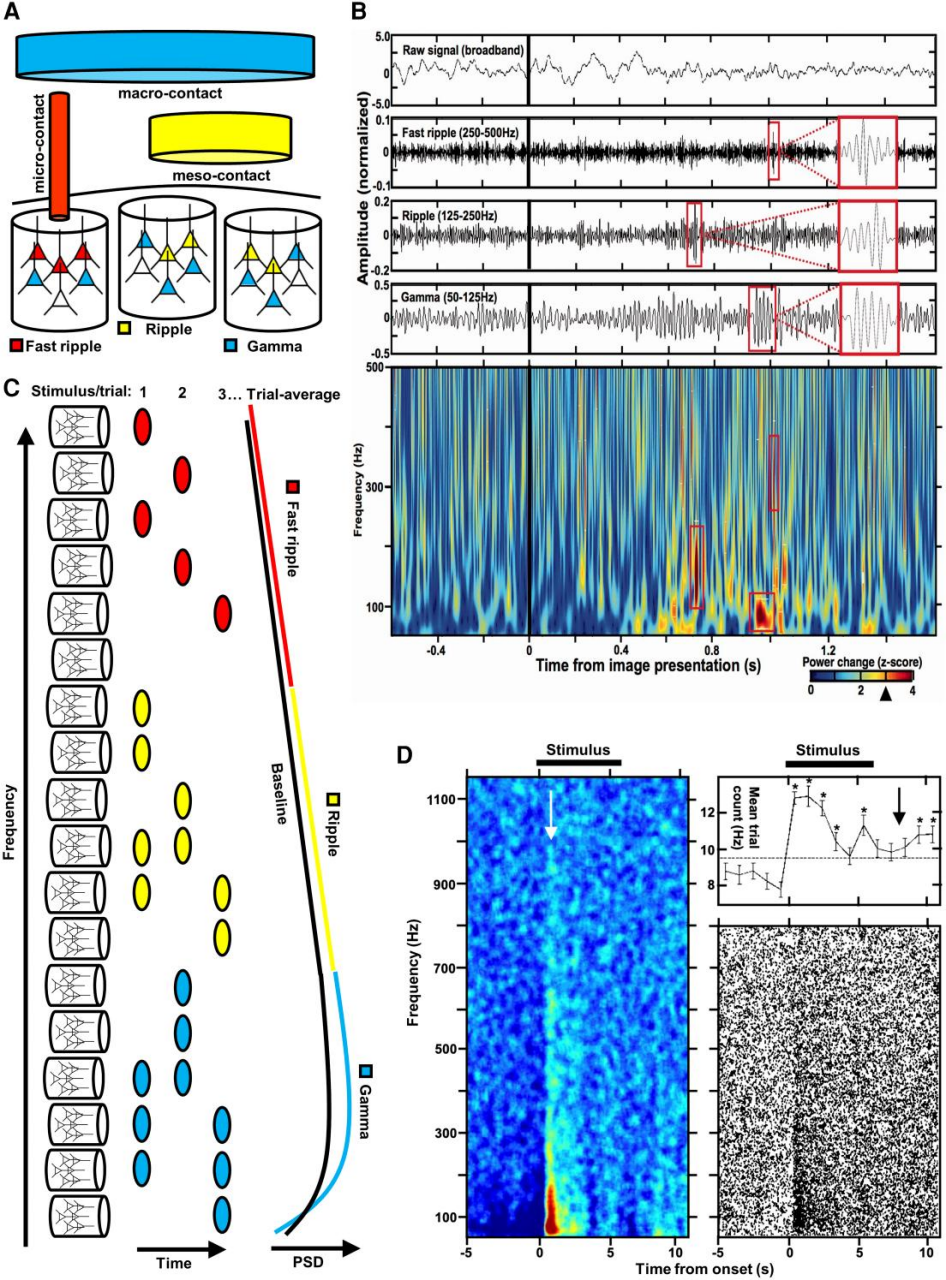
**Discrete bursts of high frequency oscillations constitute spectral power responses across high frequency ranges**. (**A**) The electrode contact type determines the cortical volume of the recorded neural ensemble generating a particular high frequency oscillation (HFO). (**B**) Example macro-contact detections of three example HFO detections (zoomed-in images in boxes) in distinct frequency ranges. (**C**) Diagram explains a broadband increase in average high frequency power by pooling constituent HFO bursts from the underlying neural assemblies. (**D**) Spectrogram of trial-averaged high frequency power induced by cognitive stimulation closely overlaps with the cumulative plot of HFO detections (dots) from all trials on the right. In the case of this prefrontal macro-electrode contact, the highest frequencies of the induced power were observed up to 1000 Hz (arrow) and the rate of induced HFO detections remained elevated even after the stimulus presentation time (arrow). Adapted from Kucewicz *et al*.^32,33^ PSD = power spectral density.

HFOs are thought to be generated by neural ensembles coordinated together at a range of local anatomical scales and network architectures, and detected in the field potentials sampled with micro-, meso- or macro-electrode contacts (Fig. 2). A smaller and more local ensemble confined to a single cortical column would generate a HFO in the higher frequencies of, for example, the fast ripple range, detected only on a single micro-contact, whereas a gamma-frequency HFO originating from a larger ensemble more widely spread across cortical columns (Fig. 3A) would be detected on several micro- and on macro-contacts.^68-70^ This gradation of anatomical scale along the frequency range of HFOs, starting from ‘macro’ oscillations on the low end through to ‘micro’ electrophysiological activity on the high end, fits into the picture of bridging network oscillations and single unit activities (Fig. 1).

On the micro-scale of single neurons, it is known that HFO generation is associated with distinct firing patterns of inhibitory interneurons that gate firing of the excitatory pyramidal cells. The original studies of ripple and fast ripple HFOs in epilepsy patients showed synchronized firing of neurons around the amplitude peaks^47,59,71^ that were found to be phase-locked to the cycle of the HFO. This coordinated firing to the phase of a given ripple HFO was observed among a large proportion of excitatory pyramidal cells,^25,29,60^ which was preceded by bursts of interneuron firing.^59^ An analogous pattern of firing has been reported in rodents^72^—pyramidal cells were spiking at the negative phase of the oscillation with the preceding inhibitory cell rhythmic firing matching the ripple frequency.

In addition to this ‘in-phase’ mechanism of generation, HFOs in the ripple and fast ripple frequency ranges can be generated through ‘out-of-phase’ population firing,^66,67^ which explains the emergent HFO frequencies beyond the limits of the refractory periods of individual neurons. Separating these different mechanisms may not be possible using spectral methods but should be feasible with high-density microelectrode recordings of the underlying neuronal spiking, as in the case of laminar multielectrode arrays across the superficial and deep cortical layers.^73^

Rodent studies using optogenetic activation or silencing of specific types of cells showed that local activation of a small group of excitatory cells is sufficient to induce ripple HFOs artificially.^74^ A similar HFO induction was achieved by locally activating parvalbumin-positive interneurons, which resulted in the phase-locking of neuronal spiking and induction of gamma oscillations.^75,76^ Hence, even though the original human studies were performed in people with epilepsy, the cellular mechanisms agree with the physiological origins unveiled in these rodent studies. Recent investigations of physiological firing patterns underlying ripple HFOs during human memory and cognitive processes^25,29,30,60^ confirm this coordinated neuronal spiking to specific phases of the oscillation.

The origins of any one HFO can, therefore, be traced to the coordinated firing of a subset of neurons. In contrast to the sharp-wave bursts, which are driven by synchronized firing across a large number of neurons in a given population, the ripple oscillation itself involves only a subpopulation of cells.^44,45,77^ The coordinated firing of the cells underlying HFOs, i.e. the neural ensembles or assemblies, can be sampled in the extracellular field potential at various spatial scales, depending on the electrode contact type used (Fig. 3A). For example, HFOs in the fast ripple range were found to be more local and confined to even a single micro-contact compared to the equivalent events detected in the ripple frequencies on two or more contacts.^64^ On the other side of the scale, oscillations in the gamma and ripple frequency ranges are commonly detected simultaneously on several micro- and macro-contacts sampling from much wider volumes of neural tissue compared to more local fast ripples.^64,68^

### HFOs are detected as bursts of spectral power in discrete frequency range and duration

Several distinct HFOs are typically detected on an electrode contact in different frequency ranges. An example is presented in Fig. 3B with four HFO detections in the gamma and epsilon/ripple frequencies. Each has a discrete duration, lasting typically between 20 and 50 ms and centred around a particular peak-amplitude frequency (marked in blue), with exception of non-oscillatory detections that span a broad range of frequencies (red). The former (oscillations) can be observed in the unfiltered raw signal, whereas the latter are associated with a sharp transition in the filtered signals (Fig. 3B). Such broadband increases of high-frequency power can result not only from the sharp transitions in the signal (e.g. interictal epileptiform spikes) but also from other sources, including muscle, eye-movement and blinking artefacts^78-82^ or neuronal firing.^39,40,83^ Individual HFO detections, on the other hand, result in a confined increase in power around a narrow span of the peak amplitude frequency. They can be described as discrete bursts of oscillation lasting at least four cycles^46,68,84^ that occur spontaneously or in response to, for example, cognitive stimulation.

The HFO bursts were first shown in the human cortex in response to the presentation of visual stimuli.^32,85^ Analogous bursts were associated with working memory performance in non-human primates.^86,87^ Most of the physiological HFO detections were found to have the same properties of discrete bursts, which were distinct from the ones with a broad frequency span,^32,33^ as in the example presented in Fig. 3B (red boxes).

### Bursts of HFO power are physiologically distinct from broadband power increase

It is important, however, to distinguish the actual bursts of physiological oscillations from other sources of increased power in the high-frequency ranges mentioned above.^23,24,34^ Spectral power across these ranges is known to generally correlate with neuronal firing.^37-40,60,81,82,88^ This firing may not necessarily be related to an actual oscillation like in the case of phase-locking of action potentials to ripple HFOs. Spikes of action potentials in a signal can result in an increase of spectral power across a wide range of frequencies, including gamma and even lower EEG bands.^83^ Although micro-contact recordings of the local field potential are especially affected by this phenomenon, given that the sharp waveforms of action potential spikes are recorded in these high-impedance signals, this so-called ‘spike-bleeding’ can also affect macro-contact recordings but may be removed by detecting broad frequency spans of increased spectral power or phase-coupling to slower oscillations.^33,83,89^

A recent study in the macaque visual cortex identified two distinct sources of increased power in the high gamma frequency range in response to light flash stimulation^90^: initial ‘early’ neuronal firing in deep cortical layers and ‘late’ dendritic field potentials in the superficial layers. This finding in non-human primates is reminiscent of the findings in the human visual cortex using word names displayed on the screen for memory encoding.^33^ Even though the human study was limited to macro-contact recordings, it could still separate an early increase in detections with a broad frequency span on individual trials, which could correspond to enhanced neuronal firing in response to stimulus presentation. This early response was followed by a later more gradual increase in HFO detections with a confined frequency span centered around the peak power of each detection. The latter oscillatory detections outnumbered the former non-oscillatory power increases in the low gamma band. This disproportion gradually decreased with more events of broad frequency span detected in the high gamma and epsilon ranges.^33^ Ripple and fast ripple frequency ranges would contain proportionally more non-oscillatory power increases, which is expected as the frequencies approach the ranges used for detecting single unit action potentials (>600 Hz). The oscillatory and non-oscillatory components can be separated based on the spectrogram characteristics of each detection.^33,83^

It does not mean, however, that action potential firing is not contributing to the oscillatory events. Both local dendritic currents and action potentials from cells as far from the recording micro-contact as >0.1 mm were found to contribute to the power of ripple oscillations in rodents.^91^ In humans, a recent careful examination of power increases in the 80–120 Hz range recorded on macro-contacts in the temporal cortex confirmed that these comprise several individual HFO bursts detected on micro-contacts, which, in turn, are related to bursts of coordinated action potential firing.^60^ On the micro-scale, these results are congruent with the original studies in human epilepsy, concluding that subsets of excitatory pyramidal cells would synchronize their spiking at specific windows of inhibitory cell firing,^47,59^ locking to a particular phase of the oscillation.

### Individual HFO bursts can be used to trace specific neural assemblies?

The results published by Tong *et al*.^60^ reconcile the previous observations that increased power across a broad range of frequencies is composed of multiple HFO bursts detected at discrete frequencies.^32,33,85^ In Figs 2 and 3, we summarize the general mechanism from micro-scale ensembles of firing neurons, through bursts of individual HFOs detected in particular trials at specific frequencies, to the resultant trial-averaged enhanced power across a broad frequency range. Coordinated firing in response to a stimulus presentation gives rise to HFOs at particular frequencies depending on the size and spread of the underlying neural ensemble (Fig. 3A and C). Other ensembles generate HFOs at particular frequencies in response to stimuli in subsequent trials. Eventually, multiple trials result in a uniform shift in power across a broad frequency range of the spectrum relative to a pre-stimulus baseline (Fig. 3C). Detections from specific trials can be displayed together as points at their corresponding peak-amplitude on a cumulative time-frequency plot, producing a pattern closely overlapping with the trial-averaged power spectrogram (Fig. 3D).

This is an explanation for the resultant broadband shift in power across the high-frequency spectrum associated with cognitive and motor tasks and increased neural firing,^92-95^ which argued against oscillations at particular frequency bands. If the intermediate step of detecting individual bursts of oscillations on a trial-by-trial basis is skipped, the overall trial-averaged power will be most highly correlated with general firing rates in the entire neural population without any common temporal pattern or coordination to oscillations. If, however, independent constituent bursts of oscillations and the underlying firing in subsets of neural ensembles are first resolved one by one, then multiple patterns of coordinated activity emerge. In this large-scale mechanism, coordinated electrical activity from multiple neural sources generating oscillations at distinct frequencies could explain the broadband shifts in power across the spectrum.^24^ Separate sources of HFO bursts detected at various frequencies remain to be demonstrated on the macro- and micro-recording scales.

Assuming that individual HFOs can indeed be separated based on their spectral features^96-98^ and thus identify particular sources of LFP activities, it should be possible to resolve the neurophysiological substrates of memory and cognition proposed in our title question. High frequency LFP activities were suggested to track particular neuronal assemblies on the level of micro-contact LFP in rodents.^91^ Intracranial recordings in non-human primates^86,87^ and in human patients^22,32,85^ can also resolve distinct bursts in the frequency-time space of individual trials, which could hypothetically be the features of particular neuronal assemblies.^24^ HFO bursts beyond the ripple frequency range, which were shown to be generated very locally on the scale of a single cortical column,^64^ would correspond to arguably the fundamental level of neural organization and information processing.^99^ In the next section, we will review the roles of temporal coordination in gamma and higher frequencies in supporting processes of memory and cognition.

## A fundamental role of high frequency oscillations in memory and cognitive processing

### Oscillations in high frequencies are pivotal to models of neuronal assemblies

The temporal coordination of neuronal firing was originally proposed to explain cognitive processing in neuropsychological theories developed by Jerzy Konorski^100^ and Donald Hebb.^101^ They envisioned that neurons that are active at the same time will develop connections and form assemblies, which inspired the famous phrase: ‘cells that fire together, wire together’ and introduced the concept of synaptic plasticity. Thus, assemblies would synaptically connect together cells that encode the same stimulus, as in case of the concept cells.^8,16-18^ One of the first pieces of experimental evidence for such temporal coordination was reported in the cat visual cortex, where neurons responding to the same visual features of the presented stimulus would synchronize their firing to provide a unified representation of the attended stimulus, leading to the idea of binding various sensory features by synchrony or temporal correlation.^102-104^ In other words, neurons that encode features of the same stimulus correlate their firing in time and form distinct assemblies, which were shown to oscillate at gamma frequencies in these pioneering experiments. The assemblies of neurons co-firing with gamma rhythmicity would also be organized into cortical columns^99^ that were particularly well mapped in the visual cortex.

These seminal research studies led to several hypotheses about the role of oscillations in the gamma frequencies that have remained a topic of interest and some controversy.^105,106^

We already mentioned the role in sensory object representation, which was also described as perceptual binding.^107^ Since these oscillations are not limited to sensory cortical areas, a more general role in integration and communication of information processing across the brain was proposed.^108-110^ Integrating information processing across the sensory and higher order association areas is especially important for cognitive functions like attention or working memory,^111^ where interplay with low frequency oscillations particularly in the theta frequency band is pivotal.^112-115^

Taken together, these hypotheses point to a fundamental role in cortical computations^116^ that presents a tangible substrate for the information processing and synaptic plasticity foreseen in the original neuropsychological theories of Konorski^100^ and Hebb.^101^ This fundamental role may extend to HFOs in general. The HFO bursts in the gamma ranges of the spectrum last approximately 50–200 ms of temporally coordinated firing activities among the underlying neural assemblies, which was proposed to constitute the basic units or ‘packets’ of cortical information encoding^117,118^ and a sufficient pattern for inducing synaptic plasticity.^119,120^ Hence, an individual HFO burst may provide a viable unitary event for cognitive processing, congruent with the proposed anatomical substrate of neuronal assemblies^121^ at the scale of a cortical column.

### Synchronous and asynchronous high frequency LFP activities need to be considered and reconciled

This fundamental role may only apply to the actual oscillatory events as opposed to other non-oscillatory sources of high-frequency power. There has been an ongoing debate whether the electrophysiological signals recorded in the gamma frequency range and above comprise temporally coordinated ‘synchronous’ or stochastic ‘asynchronous’ LFP activities.^95,97^ In the light of the aforementioned explanation of multiple discrete oscillations and their underlying distinct assemblies that together give rise to a broadband increase in power across entire neural populations (Fig. 3), the seemingly opposite views can be reconciled within the proposed large-scale frame of reference. At the micro-scale of neuronal assemblies generating individual HFO bursts, as measured in the cat visual cortex or in people with epilepsy, the temporal coordination of firing to specific phases of one oscillation can easily be detected and quantified. On the other hand, this can be much harder on the macro-scale with a multitude of assemblies each coordinated to a different oscillation. A plausible explanation proposed that there is no one global ‘clock’ or ‘metronome’ at the macro-scale of LFP activities that comprise various distinct assembly units each synchronized locally.^122^ Hence, the scale at which a given signal is recorded needs to be considered and caution applied when dissociating HFO bursts from other LFP activities.

Evoked responses to stimulus presentation are one example of non-oscillatory electrophysiological activity. Although neural mechanisms of the evoked and induced responses were shown to be related,^123^ the former is known to occur first with short latencies and ‘locked’ in time to stimulus presentation and the latter occurs at longer latencies not ‘locked’ to the stimulus. Induced responses in the EEG gamma frequency range were demonstrated by Tallon-Baudry and colleagues^124^ at around 280 ms latency from stimulus presentation and were modulated by perceptual binding of its visual features into coherent representations of illusory or real triangles, which was not observed for the evoked responses around the same latencies. A similar study was conducted with intracranial EEG recordings showing that the evoked responses were more stereotypical with a constant latency of approximately 100 ms from stimulus onset, in contrast to the induced responses in the gamma frequencies that varied from 200 to 500 ms on a trial-by-trial basis.^85^ Compared to the evoked response, the induced bursts of gamma oscillations were detected at various latencies, frequencies and amplitudes on a given trial, as shown in this and subsequent studies.^32,33,86,87^ One of these subsequent studies dissociated the actual HFO bursts from other non-oscillatory detections with a broad frequency span and found that only the latter were phase-locked to the evoked response,^33^ even though both correlated with amplitude of the evoked response potential. This confirms that the two response types are related to common neural mechanisms but are qualitatively different and separable.^123^ In contrast to the evoked response, the HFO bursts are induced at various magnitudes (related to the total number and amplitude of each detection), at a range of frequencies (around the peak amplitude of each burst), and at different latencies on any one trial depending on a given brain region.

### Spatiotemporal dynamics of high frequency power tracks cognitive processing across the brain

**Figure 4:**
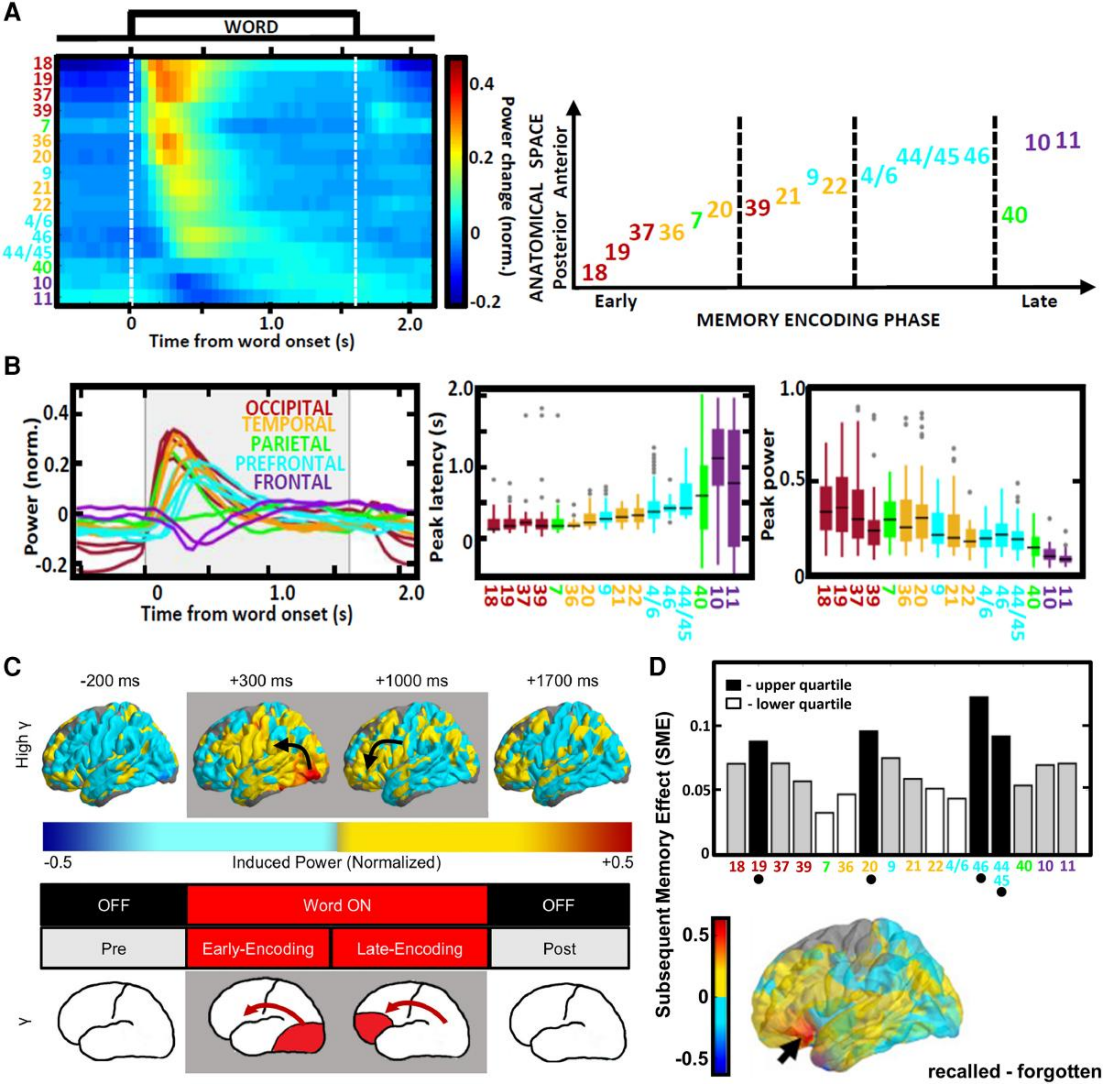
**Spectral power in the high frequency range tracks memory processing**. (**A**) Normalized high gamma/ripple frequency responses averaged from over 100 subjects around encoding of word stimuli shows a continuous wave of induced power across Brodmann cortical areas, which were sorted according to peak latency. (**B**) The earliest induction of power in the visual sensory areas reveals gradually sharper responses of greater peak power than the higher order association areas with more widely distributed and thus lower peak responses. (**C**) A wave of the induced high frequency power propagates in the posterior to anterior direction of the hierarchical processing stream of the early and late encoding phases. (**D**) The greatest differences between the power induced during encoding of the subsequently remembered and forgotten words were localized in the occipitotemporal areas of the ventral visual processing stream and in the left anterior prefrontal cortex rostral to the language-processing Broca's area. Adapted from Kucewicz *et al*.,^127^ Marks *et al*.^98^ and Topçu *et al*.^129^

Across the brain, there is a temporal pattern of the induced responses following an anatomical order of brain regions, where HFO bursts are induced along a hierarchical sequence of information processing. In response to visual stimuli, they are first induced in the primary visual areas of the occipital cortex, followed by higher order sensory processing areas in the ventral visual stream, hippocampus and amygdala, and finally induced with the longest latencies in the association areas of the temporal and prefrontal cortex.^32^ Studies that used broadband gamma power as a general index of neural activation confirmed this hierarchical sequence of induced LFP activities in the gamma frequency range,^125-128^ proceeding from an early sensory stages in the posterior anatomical areas through to late association stages all the way to the most anterior areas of the frontal pole (Fig. 4). When this broadband, trial-averaged response is visualized on a universal brain surface it gives an impression of a ‘wave’ of information processing sweeping through the cortical surface in a posterior-to-anterior direction.^98^ This ‘wave’ of high-gamma spectral power was found to be induced independent from similar waves observed earlier in the lower frequency bands^98,130^ and showed characteristic properties along the processing stream. Its amplitude was the highest in the beginning of the stream, where the latencies were the shortest with small variance, and decreased gradually toward the most anterior ends, where the latencies were the longest with a greater variance (Fig. 4B). Overall, the high-gamma power responses become less stereotypical proceeding from early to late processing in the stream, which is explained by the greater trial-by-trial variance in latencies of behavioural responses in a task.

Not all cortical areas show these induced responses—the exact localization depends on stimuli and tasks applied. The example in Fig. 4 used visually presented words as stimuli, in a task for subsequent memory recall—the identified areas with the greatest response on trials with successfully remembered words relative to the forgotten ones were localized in the visual areas processing word shapes, like the lingual gyrus of Brodmann areas 19 and 20 (Fig. 4C and D); even greater magnitude of the induced responses predicting successful memory performance were found in the language processing areas of the left lateral prefrontal cortex anterior to the Broca's field.^98,127,129^ Therefore, even this broadband power signal averaged across a wide range of high frequency LFP activities (typically 60–150 Hz) can accurately track and localize cognitive processing across the brain. It has been one of the main signals for mapping not only cognitive but also other sensory and motor processes,^24,131-133^ being highly correlated with blood oxygenation in active brain regions detected by neuroimaging methods.^134-137^ Hence, the high frequency power has been used as a general index of neuronal firing to map discrete regions involved in sensory, cognitive or motor functions.

### What cognitive processes are reflected by high frequency local field potential activities?

High frequency LFP activities can tell us more than just map areas of increased neuronal firing and blood oxygenation when processing stimuli or executing motor commands. For instance, broadband power in the high gamma frequency range induced in distinct areas of the association cortex reflects higher-order cognitive processes. It was associated with conscious visual awareness of stimuli presented in a perception task, in contrast to the same LFP activities recorded in the sensory visual areas.^138^ A similar example is the perception of dream contents during sleep.^139^ Hence, it is not only a general spectral feature of neural activation or information processing in each brain area but a signal of actual cognitive processes related to ongoing mental states.

Even in the sensory areas this signal is well known to be modulated by attention, visual awareness and vigilance states. At the fundamental level of the individual HFO bursts, a recent study in the primate visual cortex showed that the rate of bursting was modulated by the stimulus properties and attention to a given receptive field,^140^ linking ripple-frequency HFOs with goal-directed perceptual processes. The original studies in humans showed that the rate of induced HFOs was different in response to emotionally charged versus neutral or familiar versus new stimuli^22,32^ across all cortical areas. There are also more HFO bursts induced in the visual cortex on trials with stimuli that were subsequently remembered compared to the ones that were forgotten^33^—a phenomenon known as the ‘subsequent memory effect’.^141^ The occurrence and number of HFO bursts are therefore associated with memory and cognitive processes and may explain the effects observed on the level of averaged broadband power.

What process then does an individual HFO burst reflect? So far, we have established that they are generated at a micro-scale of coordinated local neural assemblies, which in case of the frequencies beyond the ripple range could be contained within volumes of a single cortical column. The bursts are induced with activation of particular brain regions in response to, e.g. sensory stimulation at various latencies and peak-amplitude frequencies. On the macro-scale of brain regions, they follow a hierarchical order or sequence of information processing and are associated with memory and cognitive functions, which modulate the rates of their occurrence. In the final part of this article, we will address the title hypothesis that the bursts reflect a basic engram process.

## A neurophysiological substrate of engram processing?

### HFOs are associated with encoding and recall of human memories

An engram is defined as a neural substrate responsible for storing and recalling a memory trace.^142^ Then, the basic requirement for an engram activity is to manifest at the time of encoding and recalling memory—there are multiple activities engaged during encoding or recall of a memory but only the ones that are consistently present in both can be specific to engram processing. If we thus follow assumptions of the Konorski^100^ and Hebb^101^ theories that neural assemblies provide feasible substrates for engrams, then their coordinated electrophysiological activities, emerging from coincident neural firing, could be traced as a feature of an engram process. HFO bursts would be a suitable candidate for such a spectral feature of coordinated firing as engrams are formed and reactivated when memories are encoded and recalled.

Our original studies in epilepsy patients^32,33^ showed that bursts of HFOs in the gamma and higher ranges are induced during encoding and recall of memorized stimuli. The studies in primates^86,87^ detected them also throughout delay phases of a working memory task, suggesting that memory traces for memorized stimuli are maintained in the form of such discrete synchronous events rather than continuous neuronal firing.^143^ Still, this is not enough to claim that an HFO burst underlies an engram activity for a specific stimulus.

A series of recent studies in epilepsy patients shed light on the hypothetical engram activity. Inspired by the role that sharp-wave ripples in rodents play in forming and retrieving memories for places,^52,53,144,145^ Norman and colleagues^28^ showed that ripple HFOs, which are induced in the hippocampus in response to presentation of images, are then reoccurring immediately preceding spontaneous recall of the remembered ones. This spontaneous (not stimulus-induced) increase in the HFO rate before recollecting a memory was specific to images that induced more bursts during encoding—only those images that were presumably ‘well’ encoded or attended were subsequently freely recalled (the subsequent memory effect) heralded by an increased rate of bursting. Although the authors did not resolve individual HFO bursts in the neocortex, they found that broadband power in the high gamma range coincided with the ripple bursts in the hippocampus and used them to match the encoding and recall patterns for specific images remembered.

Vaz and colleagues^25^ found in a similar study with epilepsy patients that neocortical HFOs in the anterior temporal lobe are underpinned by sequences of neuronal spiking, which are repeatedly detected during encoding, throughout delay, and before recall of word stimuli. The sequences of spiking, which was locked to phases of the burst oscillation, were specific to the word stimuli remembered and predicted failed memory performance when the order of a sequence was disrupted. This is the first study to show in humans that HFO bursts of sequential neuronal firing are replayed in the neocortex with encoding, maintenance and retrieval of remembered stimuli, as in the case of the rodent hippocampal sharp-wave ripple recordings.^146-150^ The authors found that these neocortical bursts of coordinated sequential firing are preceded by HFOs in the hippocampus, which explains at a finer resolution the coupling between hippocampal HFOs and neocortical high gamma power reported in the study by Norman *et al*.^27,28^ Strength of this hippocampal- neocortical HFO coupling predicted successful memory retrieval. Taken together, during memory performance HFO bursts reflect phase-locked sequential neural firing coordinated in time between hippocampus and neocortex that is specific to particular memory items and indicative of their successful retrieval.

### Memory-related HFOs are observed across widespread cortical areas during wakefulness and sleep

Since engrams are formed not only ‘online’ during memory performance but also ‘offline’ when memories are stored and consolidated, an engram activity should be detected also during resting wakefulness or sleep when they are reactivated as we think or dream about a particular memory. Coordinated hippocampal-cortical HFO bursting has now been reported also outside of task performance throughout states of wakefulness and sleep.^29,30^ Dickey and colleagues^29^ first characterized ripple HFOs detected during non-REM sleep in terms of their anatomical location in the neocortex and their detection rates, as well as the duration, peak frequency and amplitude of each detected burst. Their findings were in agreement with the previously reported detection rates and general properties like amplitude, duration, and oscillation frequency of individual cortical HFO bursts during memory performance.^32,85^

More importantly, the study provided more in-depth and detailed insight into their neuronal mechanisms and possible roles in memory consolidation. The authors detected the ripple bursts and the underlying neuronal firing during sleep and wakefulness across all cortical areas, though less densely in the association cortex. One of the previous studies showed that these general rates can be modulated by cognitive states with more bursts detected in the sensory areas during encoding of stimuli than during recall and, vice versa, more in the association areas during recall than during encoding.^32^ Dickey and colleagues did not focus their study as much on the state modulation but rather provided exquisite detail of the neurophysiological mechanisms of each burst, demonstrating phase-locking of putative pyramidal and interneuron spiking to the high-frequency oscillation, which is consistent with the previously proposed mechanism of HFO generation.^47,59,64^ This coordinated timing of neuronal firing was concluded to be ‘optimal’ for facilitating neuronal plasticity, as reported for the hippocampal sharp-wave ripples,^119,120^ and thus ideally suited to support memory and cognitive functions required for an engram activity during both ‘online’ encoding and recall, and ‘offline’ storage and consolidation of memorized information.

In a parallel study, Dickey and colleagues^30^ reported more evidence for the essential role of the hippocampal-cortical ripple bursts in the hypothetical engram processes. They report that the ripple bursts detected across multiple cortical areas or even hemispheres co-occur, phase-locking with a consistent lag. Interestingly, the neocortical bursts co-occurred with the hippocampal ripple HFOs as well but were not phase-locked to these oscillations, suggesting that the phase-locking is mediated by more direct cortico-cortical connections. This precisely synchronized burst of neural co-firing was increased preceding memory recall, making the authors suggest that their observations support the hypothesis for the role of synchrony in the retrieval of particular memories and, more generally, the role in binding perceptual and mnemonic representations.^102-104^ All in all, the burst co-firing was detected both during ‘online’ retrieval of memories and ‘offline’ during wakefulness outside of any task and in sleep, making a compelling case for a viable engram activity reflecting encoding, retrieval, as well as the storage and consolidation processes.

### The role of HFOs in the consolidation of human memories remains to be directly tested

The studies by Dickey *et al*.^29,30^ and Vaz *et al*.^25,26^ contributed unprecedented detail to the neural mechanisms for possible roles that HFO bursts could play in ‘online’ and ‘offline’ memory processing. However, none of these have directly tested their roles in the engram storage and consolidation. One of the first studies to investigate the ‘online’ and ‘offline’ role of ripple HFOs in human memory consolidation found that the hippocampal and cortical HFO bursts predicted post-sleep recall of memory items, which were encoded before sleep.^31^ This and subsequent studies confirmed that the hippocampal-cortical HFO bursts are not only coordinated with each other but are also coupled to delta slow-wave oscillations and cortical sleep spindles.^29,31,151,152^ This temporal coupling provides mechanistic evidence for the classic theories of how memories are transferred, stored and consolidated between the two structures in sleep and quiet wakefulness.^153-156^ In rodents, the hippocampal and neocortical coupling of HFO bursts was shown to be strengthened during post-learning sleep and suggested to mediate the transfer of memory traces across the two structures.^50^ Disrupting this transfer process ‘online’ or ‘offline’ with optogenetic or electrical stimulation is known to slow down learning and interfere with retrieval and consolidation processes.^51-53^ Comparable causal evidence remains to be demonstrated in humans with direct electrical stimulation to either interfere or enhance memory processing.^4,157,158^ So far, the engram processes proposed for the HFO bursts have not been directly tested in humans.

### Hypothetical neurophysiological correlates of engrams await further verification

In the animal models, the engram research has predominantly focused on the cellular and molecular substrates and mechanisms of synaptic plasticity^159-161^ with very impressive demonstrations of manipulating, silencing or even *de novo* generation of ‘artificial’ memories. Pharmacological and imaging experiments in humans corroborate similar dynamics and manipulation of engram formation and consolidation^162,163^ but evidence for analogous cellular and molecular substrates remains to be provided. How these basic cellular mechanisms relate to the electrophysiology of LFP activities remains elusive. One problem in linking the cellular mechanisms with the electrophysiological activities is that the former are spread across large neural populations distributed throughout the entire brain whereas the latter are sampled only in selected spatially confined areas of electrode contact implantation. A recent study in mice showed that almost half of all cortical and subcortical brain regions studied revealed molecular markers related to encoding of one memory trace, which were then reactivated during recall in another half of these.^164^ Hence, a quarter of the studied regions participated in the formation and reactivation of a single engram. The other quarter that was originally activated was not specific to the engram processes but related to non-specific sensory and other processes. Hence, the molecular activity was not specific to the engram processes selectively. In the same manner, HFOs can serve as a non-specific electrophysiological activity to track engram processes under particular definitions of memory encoding and recall.

Another problem is that within any one of these regions the configuration of engram cells may differ between memory encoding and recall. Electrophysiological recordings in rat hippocampus and prefrontal cortex performing a spatial working memory task showed that neural assemblies of cells firing together during encoding of either left or right lever are not the same as the assemblies reactivated during delay or recall of the lever position.^143^ Nevertheless, the large-scale recordings of HFOs can potentially provide a less variable engram signal in time and space than the molecular or the electrophysiological activity of single cells.

In an ideal scenario, there would be one assembly of engram cells that is first active during encoding of a memory and then reactivated during its maintenance and recall. That assembly would generate HFOs detected at a consistent frequency and amplitude by a recording electrode contact. However, the engram activities appear highly dynamic in terms of their spatial localization in the brain, distance from the recording contacts, and across time of memory processing. The emerging neurotechnologies are only now making it possible to investigate engram stability over longer periods of time as new cells join and leave particular neural assemblies.^121^ HFO localization was also shown to be dynamically changing with time even in the case of mapping epileptic discharges in the brain.^165^ Memory and cognitive processes would arguably turn out to be much more dynamic and distributed across the brain. Tracking these highly dynamic bursts at a large-scale of LFP activities across anatomical space of the brain and chronically over time holds promise for capturing the underlying aberrant engram assemblies.

## Conclusions, outstanding questions and future perspective

### Global sequences of HFO bursting are analogous to synfire chains of single unit spiking

In this review, we have summarized the current evidence for HFOs supporting engram processes. Oscillations in these high frequency ranges meet the basic requirements for a fundamental unitary activity that would coordinate assemblies of connected cells underlying the remembered information. They can be detected within a micro-scale of single cortical columns or on a macro-scale of multiple neighbouring electrode contacts in case of the fast ripple and gamma frequency bursts, respectively. On the micro-scale, these oscillations are aligned with spiking of neuronal assemblies that underlie encoding and recall of specific memorized stimuli. On the macro-scale, the bursts contribute to the spectral power induced across sensory and higher order association areas as the visual, semantic or affective features of the remembered stimuli are processed.

**Figure 5:**
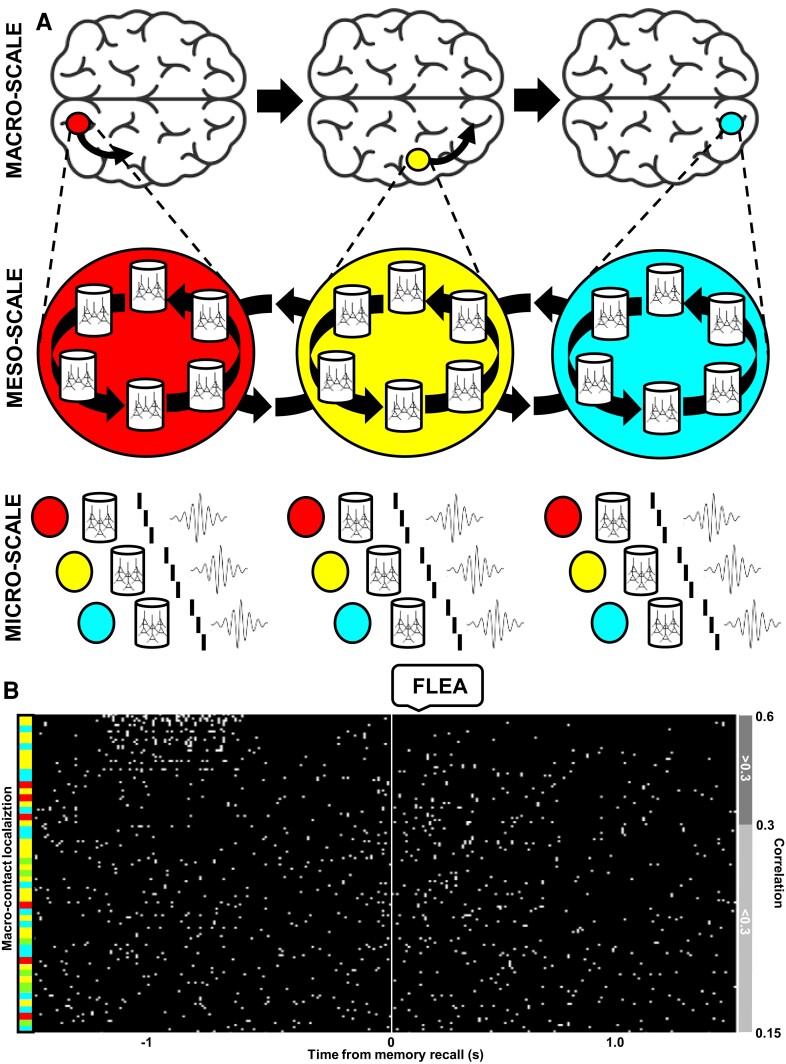
**Large-scale dynamics reconciles local and global spatiotemporal coordination of high frequency oscillation bursts across the brain**. (**A**) Schematic model of high frequency power induced in the occipital, temporal and frontal lobes that proposes a simplified mechanism of temporally coordinated high frequency oscillation (HFO) bursting between locally and globally connected neural assemblies. (**B**) Raster plot shows coordinated HFO bursting between macro-contacts (*rows*) implanted across the cortical lobes (colour-coded as in **A**) of an example patient, which were sorted in descending order of temporal correlation during free recall of the word ‘FLEA’ from memory (vertical line indicates the start of uttering the recalled word). Notice temporally coordinated bursting between the three cortical areas within approximately 1 s from the recall utterance, followed by diminished bursting activity.

This large-scale mechanism is summarized in Fig. 5A, starting with a macro-scale view of the spectral power from multiple underlying meso-scale sources, which is propagated across occipital, temporal and frontal lobes. Each of these sources can theoretically be traced to neural assemblies connected on a micro-scale of individual cells that participate in processing (and binding?) sensory, semantic and affective features of an engram. The latest HFO studies show concurrent detection of ripple frequency bursts between multiple sensory and higher order association areas in the neocortex and the hippocampus.^29,30^ These brief bursts of firing with coordinated timing across multiple areas provide an ideal substrate for supporting multisensory, abstract engram representations in the human brain and mind.

Exactly how such large-scale HFO burst dynamics would operate is an open question. One possibility is that they would form synfire chains analogous to the ones described for neuronal spiking.^166^ In this scenario, each burst could be treated like a point process analogous to an action potential^22^ but viewed on a macro-scale as in Fig. 5. HFOs detected at the same time on selected contacts from multiple cortical areas as memories are recalled (Fig. 5) would reflect sequences of spike firing observed on micro-scale of a single area.^25^ We are now positioned to test these hypothetical mechanisms in the new large-scale, high-density, human brain recordings with combined macro-, meso- and micro-electrode contacts during memory tasks. These recordings present a unique opportunity to track such basic electrophysiological activities and ascribe them to the mental processes engaged in the formation and retrieval of memory items.

### Are pathological and physiological HFOs reflecting the same underlying processes?

One challenge is that the human recordings are mostly performed in people with epilepsy, which are known to generate pathological HFOs. Distinguishing the pathological and physiological bursts has been an ongoing quest with various states of sleep, quiet wakefulness and cognitive performance proposed as viable approaches to separate them.^55-57,167-169^ Another approach is to treat both as the same process that has been ‘hi-jacked’ in epilepsy pathophysiology but, otherwise, involves common neural substrates and mechanisms. For example, we proposed that the mechanisms of memory consolidation ascribed to ripple HFOs are engaged in the process of developing neural assemblies that underlie seizure generation.^170^ Whether such ‘seizure engrams’ utilize common electrophysiological and synaptic mechanisms as those involved in memory processing remains to be established. There may be some HFO discharges that are specific only to epilepsy pathophysiology like the ultra-fast HFOs,^171^ which were found almost exclusively in the areas associated with seizure generation. Where is the upper frequency boundary for memory-related HFOs? This is another outstanding question with no clear answer so far.

### One HFO burst: one memory trace?

Finally, a key question for the engram hypothesis is whether a given HFO could track a particular memory. Would it be specific to an engram or other non-specific process? Would it be one or more neural assemblies encoding a memory or several supporting multiple related memory traces? Would concept cells participate in the assemblies and their HFO generation and make them more specific? Would each assembly generate bursts at the same frequency consistently in time and anatomical location? An assembly of a given size could in theory oscillate together at a consistent resonant frequency, providing a signature frequency for the encoded information. In practice, however, the highly dynamic nature of neural assemblies with some cells joining and some leaving,^121^ as memories are consolidated and reconsolidated over time,^172^ makes it virtually impossible to track particular engrams with HFOs. They are, arguably, still a better feature than individual spiking cells in terms of their accessibility to record over a large scale of electrophysiological signals, which are more stable and resistant to chronic biophysical changes at the recording site of an electrode contact.

Such considerations are critical for potential use as features in brain-computer interfaces^173^ to track and modulate memory processing. Our knowledge of how a given HFO can be traced to remembered stimuli is very limited compared to neuronal spiking. For example, it is known that individual concept cells can specifically encode an abstract representation^17^ or that neural populations can sparsely encode specific stimulus features^5,16^ but less is known about HFOs.^22^ Would there be a ‘core’ neural assembly that participates in encoding most stimuli of the same type, for example, words, or would there be separate assemblies for each word with some or no overlap between assemblies? These questions present testable predictions about the anatomical localization of HFOs detected on micro- or macro-scale, about their consistency of detection across stimuli and maybe even their characteristic frequencies. Predictions like that can be directly tested with brain stimulation to impair or enhance particular engrams as performed in the rodent cellular studies. The currently available experimental evidence is yet to show whether HFOs could be the neurophysiological substrate to track the human engrams.
